# TTCOV19: timing of tracheotomy in SARS-CoV-2-infected patients: a multicentre, single-blinded, randomized, controlled trial

**DOI:** 10.1186/s13054-022-04005-0

**Published:** 2022-05-18

**Authors:** Måns Eeg-Olofsson, Nina Pauli, Louise Hafsten, Josephine Jacobsson, Christopher Lundborg, Magnus Brink, Helen Larsson, Ellen Lindell, Karin Löwhagen, Magnus Gisslén, Henrik Bergquist

**Affiliations:** 1 Department of Otorhinolaryngology, Head and Neck Surgery, Institute of Clinical Sciences, Sahlgrenska Academy, University of Gothenburg, Gothenburg, Sweden; 2 Department of Otorhinolaryngology, Head and Neck Surgery, Region Västra Götaland, Sahlgrenska University Hospital, Gröna stråket 5, 413 45 Gothenburg, Sweden; 3 Department of Anesthesia and Intensive Care Medicine, Institution of Clinical Sciences, Sahlgrenska Academy, University of Gothenburg, Gothenburg, Sweden; 4 Department of Anesthesia and Intensive Care Medicine, Region Västra Götaland, Sahlgrenska University Hospital, Gothenburg, Sweden; 5 Department of Infectious Diseases, Institute of Biomedicine, Sahlgrenska Academy, University of Gothenburg, Gothenburg, Sweden; 6 Department of Infectious Diseases, Region Västra Götaland, Sahlgrenska University Hospital, Gothenburg, Sweden; 7 Department of Otorhinolaryngology, Region Västra Götaland, NU-Hospital Group, Head and Neck Surgery, Trollhättan, Sweden; 8 Department of Otorhinolaryngology, Region Västra Götaland, Södra Älvsborg Hospital, Boras, Sweden

**Keywords:** COVID-19, Mechanical ventilation, Intensive care, Time factors, Tracheotomy

## Abstract

**Background:**

Critically ill COVID-19 patients may develop acute respiratory distress syndrome and the need for respiratory support, including mechanical ventilation in the intensive care unit. Previous observational studies have suggested early tracheotomy to be advantageous. The aim of this parallel, multicentre, single-blinded, randomized controlled trial was to evaluate the optimal timing of tracheotomy.

**Methods:**

SARS-CoV-2-infected patients within the Region Västra Götaland of Sweden who needed intubation and mechanical respiratory support were included and randomly assigned to early tracheotomy (≤ 7 days after intubation) or late tracheotomy (≥ 10 days after intubation). The primary objective was to compare the total number of mechanical ventilation days between the groups.

**Results:**

One hundred fifty patients (mean age 65 years, 79% males) were included. Seventy-two patients were assigned to early tracheotomy, and 78 were assigned to late tracheotomy. One hundred two patients (68%) underwent tracheotomy of whom sixty-one underwent tracheotomy according to the protocol. The overall median number of days in mechanical ventilation was 18 (IQR 9; 28), but no significant difference was found between the two treatment regimens in the intention-to-treat analysis (between-group difference: − 1.5 days (95% CI − 5.7 to 2.8); *p* = 0.5). A significantly reduced number of mechanical ventilation days was found in the early tracheotomy group during the per-protocol analysis (between-group difference: − 8.0 days (95% CI − 13.8 to − 2.27); *p* = 0.0064). The overall correlation between the timing of tracheotomy and days of mechanical ventilation was significant (Spearman’s correlation: 0.39, *p* < 0.0001). The total death rate during intensive care was 32.7%, but no significant differences were found between the groups regarding survival, complications or adverse events.

**Conclusions:**

The potential superiority of early tracheotomy when compared to late tracheotomy in critically ill patients with COVID-19 was not confirmed by the present randomized controlled trial but is a strategy that should be considered in selected cases where the need for MV for more than 14 days cannot be ruled out.

*Trial registration* NCT04412356, registered 05/24/2020.

**Graphic abstract:**

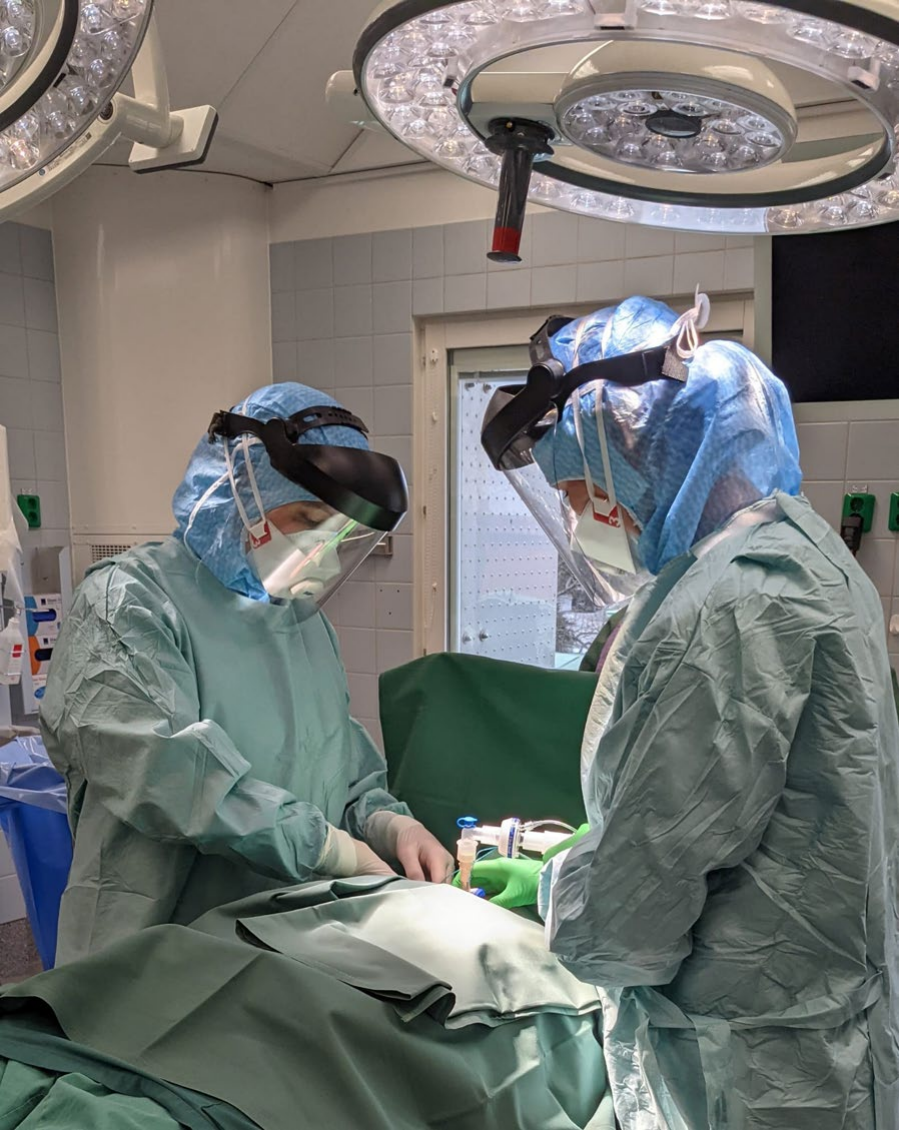

**Supplementary Information:**

The online version contains supplementary material available at 10.1186/s13054-022-04005-0.

## Background

Globally, the SARS-CoV-2 pandemic has put health care in a highly demanding situation [1]. Critically ill patients may develop acute respiratory distress syndrome (ARDS) and require respiratory support, including mechanical ventilation (MV), in the intensive care unit (ICU). After an initial period with an endotracheal tube, a tracheotomy is routinely performed to prevent potential airway complications, improve pulmonary secretion clearance, reduce the need for sedation, facilitate weaning from MV and enable speech and oral nutrition. The patient can also be nursed outside the ICU with a tracheostoma. The disadvantages of tracheotomy include early complications such as stomal infection, bleeding, subcutaneous emphysema, cannula obstruction and cannula displacement. Examples of late complications include a persistent stoma, tracheal stenosis, a tracheoesophageal fistula and granuloma formation [2].

The optimal timing of tracheotomy is debated. Several randomized, controlled trials (RCTs) that have evaluated the advantage of early versus late tracheotomy have previously been published; however, these trials did not evaluate COVID-19 patients but rather ICU patients, who represented a broad spectrum of diagnoses [3–10]. In addition, several meta-analyses on this topic have been performed, including an updated Cochrane review in 2015 [2, 11–17]. In the latter, the authors concluded that there is a lack of data of sufficient quality to define the optimal timing of a tracheotomy. Regarding COVID-19 and tracheotomy, only a few reviews or meta-analyses and no RCTs have been published. Bier-Laning et al. [18] and McGrath et al. [19] reported studies from the first wave of COVID-19 using global institutional tracheotomy protocols and a consensus working group of experts in the field. Their conclusions and recommendations were to have a conservative approach and to delay a potential tracheotomy, and according to McGrath et al. [19], a delay until at least ten days after intubation should be taken.

An important parameter when studying the optimal timing of tracheotomy is the definition of early and late tracheotomy. The variation is substantial in referred studies, with a time span for early tracheotomy between ≤ 48 h to ≤ 21 days, and for late tracheotomy between ≥ 6 days and ≥ 29 days, which induces uncertainty interpreting the results. For the present study, we chose to follow current Swedish guidelines (see “Methods” section), and in the light of the heterogeneity of early and late tracheotomy definitions, we did not see a reason to divert from those guidelines.

A recent meta-analysis concerning the optimal timing of tracheotomy in COVID-19 patients concluded that early tracheotomy, defined as 14 days from intubation or less, implied reduced number of days in MV and days spent at the ICU compared to late tracheotomy (15 days after intubation or later) [20]. No RCTs were, however, available for this meta-analysis. Newly published both prospective [21] and retrospective [22] studies indicate that early tracheotomy implies fewer days of MV and shorter length of stay at the ICU. In accordance to the findings of the latter studies, a retrospective analysis of COVID-19 patients from the first wave of the pandemic performed at our department suggested early tracheotomy to be correlated with a shorter time on MV and, consequently, a shorter ICU stay [23]. To evaluate these findings, an RCT with the primary aim of studying the timing of tracheotomy and subsequent medical consequences was initiated by our group. The hypothesis was that early tracheotomy is beneficial for critically ill patients with COVID-19 in terms of a reduced duration of MV.

## Methods

### Study design

This was a parallel, multicentre, single-blinded RCT performed in the Region Västra Götaland of Sweden (Additional file 1). The study was approved by the Swedish Ethical Review Authority (Dnr2020-02,372 + 2021–02,700) and was performed in accordance with the Declaration of Helsinki. The study is registered with Clinicaltrials.gov (NCT04412356).

### Patients

Patients were consecutively considered for inclusion according to the following criteria:

1. Adult patients (18 years or older).
2. Patients who were intubated due to real-time, reverse transcription polymerase chain reaction (RT–PCR)-verified, SARS-CoV-2 infection with ARDS according to the Berlin definition [24].
3. Patients who were hospitalized at the Sahlgrenska University Hospital in Gothenburg or at two other county hospitals within the Region Västra Götaland of Sweden (Södra Älvsborg Hospital, Borås and NU Hospital group, Trollhättan).
4. Patients in which a need for MV for more than 14 days after intubation could not be ruled out (as assessed by the team of anaesthesiologists at the ICU in agreement with the study coordinators).

Exclusion criteria were as follows:

1. Patients where a tracheotomy performed within 7 days after intubation could be life threatening due to a poor medical condition (as assessed by the team of anaesthesiologists at the ICU in agreement with the study coordinators).
2. Patients with an anatomical abnormality of the neck impeding the tracheotomy procedure (as assessed by the anaesthesiologist or otolaryngologist).
3. Patients with no informed consent.

After matching the inclusion criteria and after the exclusion criteria were used to exclude patients, the intubated patients’ next of kin were informed about the study. If informed consent from the next of kin was given, the patient was included in the study. The inclusion window was 48 h after intubation. After discharge from the ICU, the included patients were contacted to provide their own informed consent retrospectively. If feasible, patients could give their own informed consent prior to intubation. Patients were blinded to the study assignment due to the required anaesthesia while intubated and mechanically ventilated.

Demographics and baseline characteristics are shown in Tables 1 and 2. Age, sex, body mass index (BMI), COVID-19 medication, comorbidities and Simplified Acute Physiology Score 3 (SAPS3) were recorded. SAPS3 is an algorithm frequently used by anaesthesiologists to predict the mortality risk for patients presenting to the ICU by imputing a wide range of medical data, i.e. oxygenation, cardiovascular status and the Glasgow Coma Scale score, all recorded at ICU admission [25].

**Table 1:** Patient characteristics for SARS-CoV-2-infected patients randomized to early (≤ 7 days) or late (≥ 10 days) tracheotomy (intention-to-treat population) in the TTCOV19 study

	Total	Early tracheotomy	Late tracheotomy
	(*n* = 150)	(*n* = 72)	(*n* = 78)
Age, years	64.6 (11.4)	64.3 (12.4)	64.9 (10.5)
	65.5 (58;74)	65 (57.5;74.5)	65.5 (59;73)
	*n* = 150	*n* = 72	*n* = 78
Male sex	118 (78.7%)	57 (79.2%)	61 (78.2%)
Female sex	32 (21.3%)	15 (20.8%)	17 (21.8%)
Body-mass index, kg/m^2^	33.4 (19.5)	31.3 (6.3)	35.2 (26.2)
	30.6 (27.2;34.5)	30.7 (27.3;33.8)	30.8 (27.1;35.5)
	*n* = 146	*n* = 69	*n* = 77
SAPS3*	50.8 (8.0)	51.2 (8.8)	50.5 (7.2)
	50 (45;55)	50 (45;57)	50 (46;55)
	*n* = 147	*n* = 70	*n* = 77
*Covid-19 medication*			
No	134 (90.5%)	66 (91.7%)	68 (89.5%)
Remdesivir	11 (7.4%)	4 (5.6%)	7 (9.2%)
Tocilizumab	3 (2.0%)	2 (2.8%)	1 (1.3%)
*Coexisting illness*			
Heart disease^†^	34 (22.7%)	16 (22.2%)	18 (23.1%)
Hypertension	85 (56.7%)	36 (50.0%)	49 (62.8%)
Asthma	26 (17.6%)	13 (18.3%)	13 (16.9%)
Chronic obstructive pulmonary disease	8 (5.3%)	4 (5.6%)	4 (5.1%)
Obstructive sleep apnoea syndrome	12 (8.0%)	5 (6.9%)	7 (9.0%)
Diabetes	46 (30.7%)	19 (26.4%)	27 (34.6%)

Data are expressed as the mean (SD), median (IQR) or *n* (%)

*SAPS3 Simplified Acute Physiology Score 3

^†^Heart failure or coronary artery disease

**Table 2:** Patient characteristics of SARS-CoV-2-infected patients randomized to early (≤ 7 days) or late (≥ 10 days) tracheotomy (per protocol population) in the TTCOV19 study

	Total	Early tracheotomy	Late tracheotomy	*p*-value
	(*n* = 61)	(*n* = 27)	(*n* = 34)	
Age, years	63.5 (11.9)	61.7 (14.7)	65.0 (9.0)	0.29
	64 (58; 74)	62 (52; 75)	65.5 (59; 73)	
	*n* = 61	*n* = 27	*n* = 34	
Male sex	46 (75.4%)	20 (74.1%)	26 (76.5%)	
Female sex	15 (24.6%)	7 (25.9%)	8 (23.5%)	1.0
Body-mass index, kg/m^2^	36.6 (29.6)	33.4 (8.3)	39.0 (38.4)	0.61
	32.7 (28.1;35.6)	33.5 (28.4;35.7)	30.8 (27.1;35.5)	
	*n* = 59	*n* = 25	*n* = 34	
SAPS3*	50.6 (7.4)	51.2 (9.6)	50.2 (5.3)	0.63
	50 (46;54.5)	50.5 (45;58)	50 (47; 53)	
	*n* = 60	*n* = 26	*n* = 34	
*Covid-19 medication*				
No	53 (88.3%)	25 (92.6%)	28 (84.8%)	
Remdesivir	7 (11.7%)	2 (7.4%)	5 (15.2%)	0.35
Tocilizumab	0 (0%)	0 (0%)	0 (0%)	
*Coexisting illness*				
Heart disease^†^	12 (19.7%)	4 (14.8%)	8 (23.5%)	0.60
Hypertension	35 (57.4%)	15 (55.6%)	20 (58.8%)	1.00
Asthma	8 (13.1%)	4 (14.8%)	4 (11.8%)	1.00
Chronic obstructive pulmonary disease	1 (1.6%)	0 (0.0%)	1 (2.9%)	1.00
Obstructive sleep apnoea syndrome	9 (14.8%)	4 (14.8%)	5 (14.7%)	1.00
Diabetes	17 (27.9%)	8 (29.6%)	9 (26.5%)	1.00

Data are expressed as the mean (SD), median (IQR) or *n* (%)

*SAPS3 Simplified Acute Physiology Score 3

^†^Heart failure or coronary artery disease

### Randomization

The patients who were included underwent randomized stratification with the allocation to either early (seven days after intubation or less) or late tracheotomy (ten days after intubation or more) in groups of four depending on sex and age (male < 65 years, male ≥ 65 years, female < 65 years and female ≥ 65 years). The reason for the different strata was because there was an anticipated irregularity in the cohort with a predominance of males, 65 years of age or older. An independent statistician (Statistiska Konsultgruppen AB) provided the random allocation sequence using a computerized algorithm. The participants were enrolled and assigned to interventions by the study coordinators (MEO, NP, LH and HB). A tracheotomy was performed with the intention of following the results of the randomization. To increase the protocol compliance, the randomization result was recorded in the patient record, and the anaesthesiologist in charge was informed. Moreover, a specific referral to the Department of Otorhinolaryngology was requested at the time of inclusion for patients randomized to early tracheotomy to promote monitoring of the patient. A power analysis with a target of 80% power and *p* < 0.05 (sample size of 180 patients, mean difference of 3.0 days, and SD of 6.2) was performed using data from two RCTs similar to the current trial regarding the primary endpoint; however, these studies had more heterogeneous study populations [4, 10]. Our analysis resulted in the required inclusion of 180 patients (90 patients in each randomization arm).

### Procedures

During the study period, a specific team of nine experienced otolaryngologists were assigned to perform all open surgical COVID-19 tracheotomies, while various anaesthesiologists performed the percutaneous tracheotomies. Established safety procedures and equipment were used to avoid the contamination of the staff treating the infected patient. The details of the tracheotomy procedures as well as other special considerations for the COVID-19 surgical procedures have been previously described [23].

### Outcomes

The primary aim was to explore the optimal timing of tracheotomy in relation to the need for MV, specifically to compare early tracheotomy (seven days after intubation or less) and late tracheotomy (ten days after intubation or more) regarding the total number of days needed for MV (primary endpoint). The secondary outcomes included the total number of days that patients were in need of sedation, total number of days in the ICU, total number of days from intubation to tracheotomy, type of tracheotomy, need for reintubation, mortality in the ICU, and mortality within 90 days of intubation and complications. The definition of early and late tracheotomy was decided based on the “Swedish national recommendations for tracheotomy” (https://lof.se/patientsakerhet/vara-projekt/nationella-rekommendationer-for-trakeotomi/rekommendationer-och-rad) regarding the time span for which tracheotomies are generally recommended. The three-day span between early and late tracheotomy was regarded as not too short to hide differences and not too long to induce an increased risk of protocol deviations.

### Interim analysis

An independent Data and Safety Monitoring Board (DSMB) was consulted for the safety and futility of the study (conditional power) after 50% of the patients had been included in the trial and were evaluable. This interim analysis was performed according to both the ITT and the PP principles. The safety parameters included any serious complications due to the tracheotomy procedure and death during ICU care. The futility parameters were the number of MV days after intubation and any statistical evidence that further inclusion would not change the result of the primary endpoint for either treatment arm.

### Data collection

Data were collected on case report forms (CRFs) at the time of inclusion and at discharge from the ICU, and the collected data comprised basic data concerning the patient’s COVID-19 disease, general health and the reason for intubation, timing from intubation to tracheotomy, weaning from MV, sedation, tracheotomy-associated complications, and eventual death in the ICU or death within 90 days from intubation. The CRF data were manually transferred to a master protocol file. Missing data were searched for and added if found. The primary endpoint was verified twice and independently by two of the authors. Supplementary data were verified once and complemented with additional spot checks for all data in every 15th patient.

### Statistics

All analyses that included comparisons between the two randomization groups were performed in the ITT population and the PP population. No crossover methodology was used to increase the number of patients in the per-protocol (PP) analysis. For the unadjusted comparisons between the two groups, Fisher’s nonparametric permutation test was used for continuous variables, Fisher’s exact test was used for dichotomous variables and Pearson’s Chi-square test was used for nonordered categorical variables. For continuous variables and dichotomous variables, the data were described with the mean differences with 95% confidence intervals between the two groups, along with the *p*-value. Adjusted analyses for continuous outcome variables were performed with analysis of covariance. The correlations were analysed with Spearman rank correlation.

Sensitivity analyses were adjusted for stratification variables, including age and sex, and were performed for the primary analysis and for selected secondary analyses. The distribution of continuous variables is expressed as the mean (SD) and median (IQR). Categorical variables are described by numbers and percentages. No imputation of missing values was performed. The study centre was not adjusted for since the study centres were located within the same region sharing similar regimes and, further, that some patients most likely would be transported between the different centres.

All tests were two-tailed and were conducted at the 0.05 significance level. All analyses were performed by using SAS® v9.2 (Cary, North Carolina, USA).

## Results

**Fig. 1:**
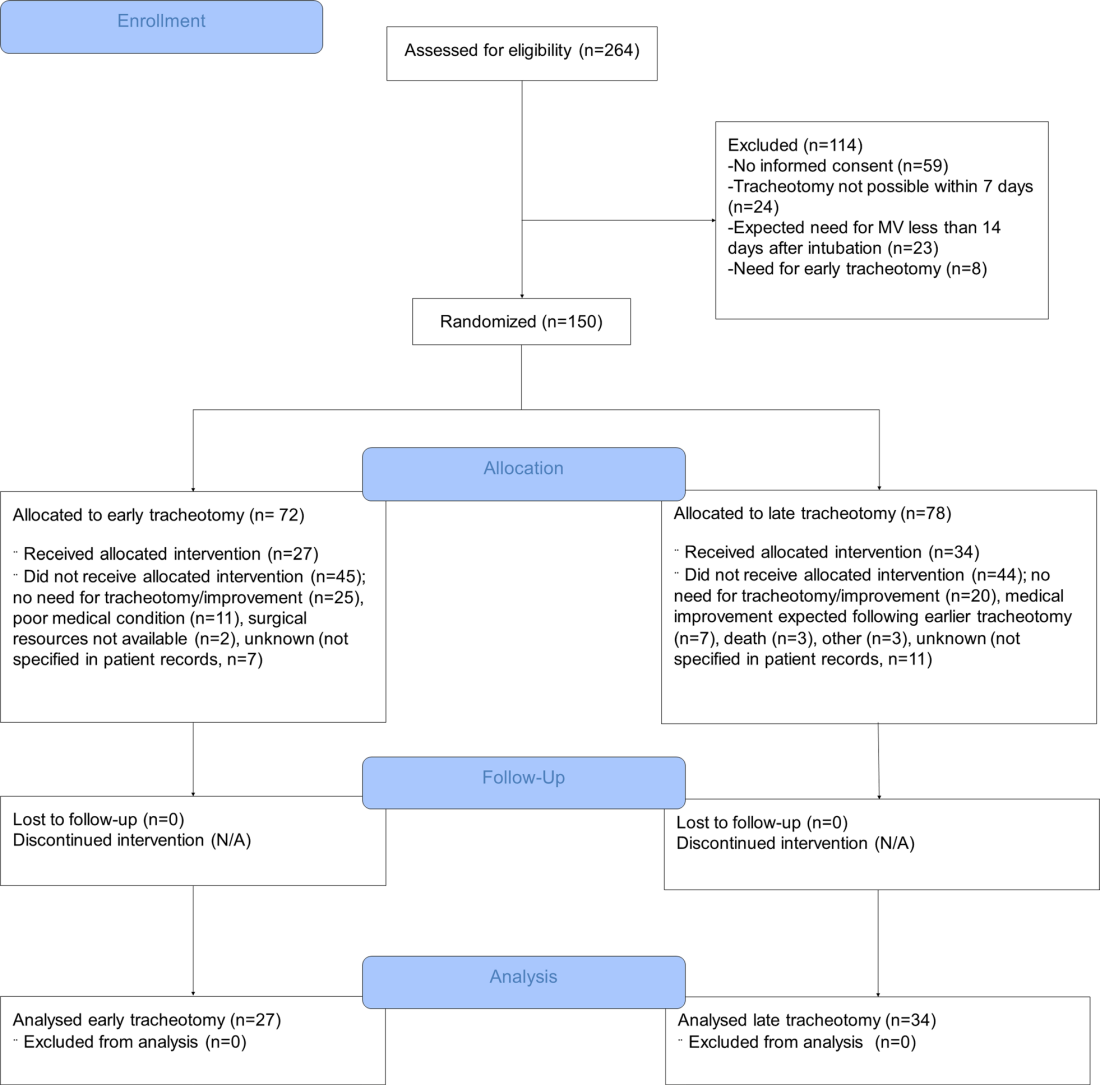
Trial flowchart for the TTCOV19 study

Between June 6, 2020, and April 20, 2021, 264 patients were consecutively assessed for eligibility to participate in the trial (Fig. 1). Of these, 114 were excluded and the most common reason for exclusion was the absence of informed consent (*n* = 59).

When 90 patients were included and determined to be evaluable (90 days after inclusion), the DSMB recommended stopping the inclusion of patients after analysing the safety and futility parameters. The interim analysis showed that there were no differences between the two arms regarding death in the ICU or serious tracheotomy complications. The ITT analysis showed no statistical significant difference between early and late tracheotomy regarding days of MV; however, there was a statistically significant difference (*p* = 0.014) in favour of the early tracheotomy group in the PP analysis. The DSMB judged these findings to be stable even if 180 patients were included. At the time of the DSMB recommendation, a total of 150 patients had been included and in conjunction with a substantial decrease in the inflow of new eligible patients, the steering committee decided to end the inclusion. Eighty percent (*n* = 120) of the patients were included from the Sahlgrenska University Hospital in Gothenburg, and 20% (*n* = 30) were included from the other two hospitals in the Region Västra Götaland of Sweden. Seventy-two patients were assigned to early tracheotomy, and 78 patients were assigned to late tracheotomy (Fig. 1).

Of the 102 patients (68%) who underwent tracheotomy, sixty-one patients (60%) did so according to the results of the randomization. Twenty-seven patients were included in the early tracheotomy arm, and 34 patients were included in the late tracheotomy arm. Common reasons for not following the intention-to-treat analysis are listed as follows: when patients no longer had an indication for a tracheotomy, when patients were in a poor general condition for surgical intervention, and when patients had a need for an early tracheotomy due to their medical condition. Of 25 patients who were randomized to early tracheotomy but did not undergo the operation within 7 days due to a rapid improvement, sixteen patients (64%) still underwent tracheotomy at a later time due to subsequent deterioration.

The demographics and baseline characteristics in the ITT and PP populations, including age, sex, BMI, COVID-19 medications given, comorbidities and SAPS3, were equally distributed and comparable between the two treatment arms (Tables 1, 2). Men were the majority among the included patients (78.7%). The median age of the whole population was 65.5 years (min/max 20; 84). The overall median number of days in MV was 18 (IQR 9; 28), but no significant difference was found for the primary endpoint between the two treatment regimens in the intention-to-treat analysis (between-group difference: − 1.5 days (95% CI − 5.7 to 2.8; *p* = 0.5) (Table 3). The correlation between the timing of tracheotomy and the primary endpoint for all tracheotomized patients (*n* = 102) was, however, significant (Spearman’s correlation of 0.39, *p* < 0.0001). In the PP analysis, there was a statistically significant mean difference in MV days between the treatment arms of − 8.03 days (95% CI − 13.85 to − 2.27; *p* = 0.0064) for the whole group and − 7.73 days (95% CI − 14.33 to − 1.13; *p* = 0.022) for survivors only, in favour of patients allocated to the early tracheotomy group. The secondary efficacy analyses for the ITT and PP population are displayed in Tables 4 and 5.

**Table 3:** Primary outcome in the TTCOV19 study

	Total	Early tracheotomy	Late tracheotomy	*p*-value	Difference between groups
Total number of days in mechanical ventilation (ITT)	20.4 (13.2)	19.6 (12.6)	21.1 (13.9)	0.5	− 1.52 (− 5.74; 2.81)
	18 (9;28)	16.5 (9;27.5)	20 (8;30)		
	*n* = 150	*n* = 72	*n* = 78		
Total number of days in mechanical ventilation (PP)	26.7 (11.8)	22.3 (11.9)	30.3 (10.6)	0.0064	− 8.03 (− 13.85; − 2.27)
	25 (18;34)	18 (12;30)	27 (22;36)		
	*n* = 61	*n* = 27	*n* = 34		
Total number of days in mechanical ventilation (PP survivors)	25.2 (10.2)	21.4 (9.8)	29.2 (9.3)	0.022	− 7.73 (− 14.33; − 1.13)
	24 (18;31)	19 (14;28)	26 (22;35)		
	*n* = 35	*n* = 18	*n* = 17		

Total number of days with mechanical ventilation for patients with early (≤ 7 days) versus late (≥ 10 days) tracheotomy, according to intention-to-treat (ITT), per-protocol (PP) and for patients who followed the study protocol and survived ICU care (PP survivors)

Data are expressed as the mean (SD), median (IQR) or *n* (%)

**Table 4:** Secondary outcomes in the TTCOV19 study for the intention-to-treat population

	Intention-to-treat population (*n* = 150)	Early tracheotomy (*n* = 72)	Late tracheotomy (*n* = 78)	*p*-value	Difference between groups Mean (95% CI)
Total number of days with sedation*	18.4 (11.8) 16.5 (9; 26) *n* = 146	17.6 (10.7) 14 (9; 23) *n* = 71	19.2 (12.7) 17 (8; 27) *n* = 75	0.42	− 1.59 (− 5.44; 2.29)
Total number of days in the intensive care unit	23.9 (15.1) 21 (12; 32) *n* = 148	23.7 (15.3) 19 (12; 32) *n* = 71	24.2 (14.9) 22 (12; 32) *n* = 77	0.83	− 0.546 (− 5.474; 4.289)
Days from intubation to tracheotomy	9.45 (4.08) 9 (7; 11) *n* = 102	7.69 (3.31) 7 (5; 9) *n* = 49	11.1 (4.1) 11 (8; 13) *n* = 53	< .0001	− 3.38 (− 4.85; − 1.92)
Total number of days with sedation during the first 28 days	16.7 (8.5) 16.5 (9; 26) *n* = 146	16.2 (8.0) 14 (9; 23) *n* = 71	17.1 (8.9) 17 (8; 27) *n* = 75	0.52	− 0.910 (− 3.697; 1.886)
Total number of days in the intensive care unit during the first 28 days	19.5 (8.2) 21 (12; 28) *n* = 148	19.1 (8.1) 19 (12; 28) *n* = 71	19.9 (8.4) 22 (12; 28) *n* = 77	0.55	− 0.809 (− 3.526; 1.844)
Total number of days with mechanical ventilation during the first 28 days	17.7 (8.8) 18 (9; 28) *n* = 150	17.2 (8.5) 16.5 (9; 27.5) *n* = 72	18.1 (9.1) 20 (8; 28) *n* = 78	0.57	− 0.842 (− 3.639; 2.083)
Need of reintubation	19 (12.8%)	11 (15.5%)	8 (10.3%)	0.48	5.2 (− 6.9; 17.4)
Death in the intensive care unit	49 (32.7%)	20 (27.8%)	29 (37.2%)	0.29	− 9.4 (− 25.6; 6.8)
Days from intubation to death	26.3 (15.6) 22.5 (13.5; 39) *n* = 52	25.6 (16.5) 18 (11; 39) *n* = 22	26.9 (15.2) 24.5 (17; 38) *n* = 30	0.78	− 1.31 (− 10.00; 7.36)
*Type of tracheotomy*					
Open surgical	82 (81%)	40 (82%)	42 (81%)		
Percutaneous	19 (19%)	9 (18%)	10 (19%)	0.99	
*Complications*					
Any complication	114 (76.0%)	53 (73.6%)	61 (78.2%)	0.64	− 4.6 (− 19.6; 10.4)
Pulmonary embolism	17 (11.3%)	4 (5.6%)	13 (16.7%)	0.056	− 11.1 (− 22.3; 0.0)
Pneumothorax	13 (8.7%)	6 (8.3%)	7 (9.0%)	1.00	− 0.6 (− 11.0; 9.7)
Ventilator associated pneunomia	6 (4.0%)	3 (4.2%)	3 (3.8%)	1.00	0.3 (− 7.3; 7.9)
Airway obstruction	13 (8.7%)	9 (12.5%)	4 (5.1%)	0.19	7.4 (− 3.0; 17.8)
Tracheal bleeding	23 (15.3%)	11 (15.3%)	12 (15.4%)	1.00	− 0.1 (− 13.0; 12.8)
Renal failure	23 (15.3%)	10 (13.9%)	13 (16.7%)	0.81	− 2.8 (− 15.6; 10.1)
Stroke	5 (3.3%)	4 (5.6%)	1 (1.3%)	0.32	4.3 (− 2.9; 11.5)
Critical illness myopathy	15 (10.0%)	5 (6.9%)	10 (12.8%)	0.36	− 5.9 (− 16.7; 4.9)
Delirium	26 (17.3%)	13 (18.1%)	13 (16.7%)	0.99	1.4 (− 12.1; 14.9)
Decubital ulcers	35 (23.3%)	15 (20.8%)	20 (25.6%)	0.62	− 4.8 (− 19.6; 10.0)
Other complications	41 (27.3%)	21 (29.2%)	20 (25.6%)	0.76	3.5 (− 12.1; 19.1)

Data are expressed as the mean (SD), median (IQR) or *n* (%)

Early tracheotomy ≤ 7 days after intubation and late tracheotomy ≥ 10 days after intubation

*Data for sedation were missing for four patients

**Table 5:** Secondary outcomes in the TTCOV19 study for the per-protocol population

	Per protocol population	Early tracheotomy	Late tracheotomy	*p*-value	Difference between groups
	(*n* = 61)	(*n* = 27)	(*n* = 34)		Mean (95% CI)
Total number of days with sedation*	23.8 (9.9)	19.7 (9.4)	27.3 (9.1)	0.0025	− 7.61 (− 12.50; − 2.88)
	22 (17,31)	18 (12;29)	26 (19.5;33)		
	*n* = 59	*n* = 27	*n* = 32		
Total number of days in the intensive care unit	30.2 (13.9)	26.5 (15.2)	33.1 (12.3)	0.059	− 6.67 (− 13.64; 0.23)
	29 (22;37)	24 (14;35)	29.5 (24;40)		
Days from intubation to tracheotomy	9.77 (4.73)	5.48 (1.37)	13.2 (3.5)	< .0001	− 7.69 (− 9.12; − 6.33)
	10 (6;12)	6 (4;7)	12 (11;14)		
Total number of days with sedation during the first 28 days*	21.4 (6.6)	18.3 (7.3)	24.0 (4.5)	0.0006	− 5.70 (− 8.81; − 2.57)
	22 (17; 28)	18 (12; 28)	26 (19.5; 28)		
	*n* = 59	*n* = 27	*n* = 32		
Total number of days in the intensive care unit during the first 28 days	23.9 (5.7)	21.2 (7.1)	26.0 (3.1)	0.0018	− 4.81 (− 7.50; − 2.08)
	28 (22; 28)	24 (14; 28)	28 (14; 28)		
Total number of days with mechanical ventilation during the first 28 days	22.6 (6.3)	19.5 (7.4)	25.1 (3.7)	0.0006	− 5.61 (− 8.50; − 2.69)
	25 (18; 28)	18 (12; 28)	27 (22; 28)		
Need for reintubation	7 (11.5%)	2 (7.4%)	5 (14.7%)	0.64	− 7.3 (− 24.8; 11.2)
Death in the intensive care unit	26 (42.6%)	9 (33.3%)	17 (50.0%)	0.3	− 16.7 (− 40.3; 9.0)
Days from intubation to death	30.3 (15.3)	23.9 (15.9)	33.6 (14.4)	0.12	− 9.67 (− 22.83; 2.20)
	28 (18;39)	13 (11;34)	28.5 (24;41)		
	*n* = 27	*n* = 9	*n* = 18		
*Type of tracheotomy*					
Open surgical	52 (85.2%)	24 (88.9%)	28 (82.4%)		
Percutaneous	9 (14.8%)	3 (11.1%)	6 (17.6%)	0.47	
*Complications*					
Any complication	55 (90.2%)	23 (85.2%)	32 (94.1%)	0.46	− 8.9 (− 28.1; 7.8)
Pulmonary embolism	9 (14.8%)	1 (3.7%)	8 (23.5%)	0.063	− 19.8 (− 38.1; − 1.9)
Pneumothorax	7 (11.5%)	3 (11.1%)	4 (11.8%)	1	− 0.7 (− 17.9; 18.4)
Ventilator associated pneumonia	3 (4.9%)	1 (3.7%)	2 (5.9%)	1	− 2.2 (− 17.1; 14.3)
Airway obstruction	9 (14.8%)	6 (22.2%)	3 (8.8%)	0.27	13.4 (− 5.3; 34.5)
Tracheal bleeding	14 (23.0%)	6 (22.2%)	8 (23.5%)	1	− 1.3 (− 23.1; 21.3)
Renal failure	10 (16.4%)	3 (11.1%)	7 (20.6%)	0.52	− 9.5 (− 28.6; 11.1)
Stroke	2 (3.3%)	1 (3.7%)	1 (2.9%)	1	0.8 (− 12.1; 16.3)
Critical illness myopathy	8 (13.1%)	1 (3.7%)	7 (20.6%)	0.11	− 16.9 (− 34.8; 1.2)
Delirium	15 (24.6%)	6 (22.2%)	9 (26.5%)	0.94	− 4.2 (− 26.5; 18.9)
Decubital ulcers	22 (36.1%)	8 (29.6%)	14 (41.2%)	0.51	− 11.5 (− 35.1; 14.3)
Other complications	20 (32.8%)	9 (33.3%)	11 (32.4%)	1	1.0 (− 23.0; 25.2)

Data are expressed as the mean (SD), median (IQR) or *n* (%)

Early tracheotomy ≤ 7 days after intubation and late tracheotomy ≥ 10 days after intubation

*Data for sedation were missing for two patients

There were no adverse events during the tracheotomy procedures of which the majority (80%) were performed with open surgical technique. Common reasons for choosing open surgical technique rather than percutaneous were high BMI, high doses of anticoagulants used and/or an effort from otorhinolaryngologists to reduce the workload for anaesthesiologists. There were no significant differences between the early tracheotomy group and late tracheotomy group regarding the type of tracheotomy, total number of days in the ICU, need for reintubation, days from intubation to death in the ICU, death within 90 days, and complications when analysed according to both the ITT and PP principles. The ICU mortality rate for the whole population was 32.7% (*n* = 49). Three patients died after being discharged from the ICU during the first 90 days after intubation. The percent of missing data not included in the calculations was 0.4%.

## Discussion

To the best of our knowledge, this is the first RCT performed in critically ill COVID-19 patients with MV where the primary aim was to clarify the optimal timing for a tracheotomy. Our hypothesis, based on previous studies, that early tracheotomy is beneficial in terms of days in MV could not be confirmed in the main analysis, i.e. according to ITT. The unpredictable course of the disease contributed to the considerable lack of compliance with the random allocation. However, in patients who followed the study protocol a significantly reduced number of days in MV was found for the early tracheotomy arm compared to the late tracheotomy arm. In addition, a strong correlation between early tracheotomy and fewer MV days was found for all patients who underwent a tracheotomy regardless of the results of the randomization.

The optimal timing for tracheotomy has been debated for many years without any clear consensus [17]. When focusing on RCTs, the previously published studies are heterogeneous in many aspects, including the definitions of early and late tracheotomy, primary endpoints, patient populations (e.g. neurological, surgical, postcardiac surgery) and results based on ITT or PP populations. These differences are likely the reason for the diverging results and the difficulty of drawing any firm conclusions. When summarizing results from frequently cited RCTs within the field and with a study population *n* ≥ 100, early tracheotomy seems to reduce the number of MV days and the ICU stay compared to late tracheotomy [4–7, 10]. However, these findings were not supported by Trouillet et al. [8] and Young et al. [9], where a substantial proportion of patients in their late tracheotomy groups were extubated and therefore not tracheotomized. Likewise, the incidence of ventilator-associated pneumonia was lower in the early tracheotomy group in some studies [5, 6, 10] but not in others [7, 8]. A reduction in the number of days that patients needed sedation was shown for the early tracheotomy group in the majority of studies where this parameter was a secondary outcome [4, 6, 8, 10]. Mortality, however, does not seem to be influenced by the timing of tracheotomy, either in the long or short term.

The mechanisms explaining why early tracheotomy patients would require fewer days of MV are speculative. There are some general advantages of tracheostoma compared to endotracheal tubes that are already described in the Introduction, although these parameters are not necessarily dependent on early or late tracheotomy. Continuous sedation is, however, associated with numerous side effects, including bradycardia, hypotension, renal failure, respiratory depression and impaired cognition [26]. It is likely that tracheotomy is associated with a reduced need for sedatives compared to endotracheal tube treatment, which is also in line with the PP results of the current study showing that early tracheotomy implies fewer days of sedation compared to late tracheotomy. With less sedation patients have a higher level of awareness, including autonomous respiration, swallowing and communication, which can facilitate the weaning of patients off of MV [27]. In addition, COVID-19 patients in the ICU have been found to have a high incidence of critical illness polyneuropathy and critical illness myopathy [28], which are more easily avoided without sedation and enabling the early mobilization of patients. In concordance with this, there was a higher incidence of critical illness myopathy in the late tracheotomy group, although the difference was not statistically significant. A higher incidence of pulmonary embolism was found in the late tracheotomy group, and this finding was also not statistically significant.

Predicting the time needed for MV in each patient is difficult for the anaesthesiologist in charge, and other authors have described a similar dilemma [4, 9, 10]. The fact that 64% of the patients who did not undergo tracheotomy within 7 days from intubation, although they were allocated to the early tracheotomy arm, still underwent the procedure later due to subsequent deterioration illuminates this problem.

With the newly gained knowledge of the clinical features of the disease, new treatment strategies have been developed during the entire COVID-19 pandemic. In the early phase, anticoagulants, corticosteroids and the advantages of prone positioning became standard treatment and were judged not to have an impact on the outcomes of early or late tracheotomy. Chloroquine treatment was tried during the early phase of the pandemic but had discouraging results, and chloroquine treatment was not used for the patients in this study. COVID-19 medications, such as Remdesivir and Tocilizumab, were in the Region Västra Götaland of Sweden introduced later in the pandemic and were therefore recorded in our study due to the potential influence on the outcomes of early or late tracheotomy. However, there was no indication of bias in either of the treatment arms.

Despite vaccination programs, the COVID-19 pandemic at the time of writing this article is still spreading globally. Although still not confirmed, implementation of early tracheotomy may limit the number of days that patients need MV, may decrease the ICU stay and could possibly decrease the severe and frequent long-term sequelae that are reported and that many patients suffer from after hospitalization from severe COVID-19 infection [29]. Moreover, it seems fair to assume that the same reasoning is applicable in patients with ARDS of other aetiologies. This statement is based on recent publications suggesting that there are negligible clinical differences between ARDS secondary to COVID-19 and ARDS not related to COVID-19 and that the damage to the mechanics of the respiratory system is similar between the two [30–32]. However, differences have been described between the two, and future studies are needed to confirm such an assumption [33].

### Strengths and limitations

The prospective, randomized controlled design of the present study, in a homogeneous patient cohort in need of MV due to severe COVID-19, is an obvious strength and is a consequence and continuation of the preliminary results from a retrospective study previously conducted at our institution [23]. The two arms (early versus late tracheotomy) consisted of comparable groups in both the ITT and PP populations, i.e. the parameters that were included, excluding the timing of tracheotomy (age, sex, BMI, SAPS3 and comorbidity), did not seem to affect the primary outcome. Additionally, the amount of missing data was low, and the recorded parameters were double- or triple-checked.

In contrast to the results for the patients who followed the study protocol, the superiority of early tracheotomy could not be statistically verified in the ITT analysis. This may have several reasons, including the risk of a type 2 error, i.e. the possibility that not enough patients were included in the trial. Notably, over 1000 patients in each arm would be required to obtain statistical significance according to a retrospective power calculation (80% and *p* < 0.05). This number of included patients would not have been feasible in our setting, and additionally, it is unlikely that the somewhat premature stopping of the study altered the main findings. The reasons for not complying with the assigned randomization were associated with the unpredictable medical course of COVID-19 in the majority of cases, including the development of a prompt recovery, severe deterioration including death or a tracheotomy performed in advance to achieve clinical progress and decrease sedation as assessed by the anaesthesiologist. With the current knowledge of COVID-19, more detailed data regarding the severity of ARDS might have added information to further ensure comparable groups for early and late tracheotomy. Nevertheless, while of inferior scientific impact compared to the ITT analysis, the results of the PP analysis are not futile and could reflect the treatment effect under optimal conditions and thereby which of the two strategies, at least in theory, to aim for. The fact that only three hospitals and in the same region of Sweden participated in the study might limit the generalizability of the results globally. The skewed distribution with a majority of patients (80%) included at the Sahlgrenska University Hospital is, however, not judged to introduce any biased selection of concern.

## Conclusions

The potential superiority of early tracheotomy compared to late tracheotomy in critically ill patients with COVID-19 was not confirmed by the present study. Nevertheless, based on our results and previous studies, early tracheotomy is a strategy that should be considered in selected cases where a need for MV for more than 14 days cannot be ruled out. Further studies are warranted to raise the level of evidence and increase the generalizability within this topic.

## Supplementary Information

**Additional file 1**. TTCOV19 Research Protocol.

## Data Availability

The datasets used and/or analysed during the current study are available from the corresponding author on reasonable request. Information of the study can also be reached at https://www.researchweb.org/is/vgr/project/276832 (Swedish). On request the corresponding author can provide Swedish language data in English.

## Abbreviations

COVID-19: Coronavirus disease 2019
SARS-CoV-2: Severe acute respiratory syndrome coronavirus 2
SD: Standard deviation
CI: Confidence interval
ARDS: Acute respiratory distress syndrome
MV: Mechanical ventilation
ICU: Intensive care unit
RCT: Randomized controlled trial
RT-PCR: Reverse transcription polymerase chain reaction
BMI: Body mass index
SAPS3: Simplified acute physiology score 3
DSMB: Data and safety monitoring board
ITT: Intention-to-treat
PP: Per-protocol
CRF: Case report form
IQR: Inter quartile range

## References

[1] Griffin KM, Karas MG, Ivascu NS, Lief L (2020). Hospital preparedness for COVID-19: a practical guide from a critical care perspective. Am J Respir Crit Care Med.

[2] De Leyn P, Bedert L, Delcroix M, Depuydt P, Lauwers G, Sokolov Y, Van Meerhaeghe A, Van Schil P (2007). Tracheotomy: clinical review and guidelines. Eur J Cardio Thorac Surg Off J Eur Assoc Cardio Thorac Surg.

[3] Barquist ES, Amortegui J, Hallal A, Giannotti G, Whinney R, Alzamel H, MacLeod J (2006). Tracheostomy in ventilator dependent trauma patients: a prospective, randomized intention-to-treat study. J Trauma.

[4] Diaz-Prieto A, Mateu A, Gorriz M, Ortiga B, Truchero C, Sampietro N, Ferrer MJ, Mañez R (2014). A randomized clinical trial for the timing of tracheotomy in critically ill patients: factors precluding inclusion in a single center study. Crit Care (Lond, Engl).

[5] Koch T, Hecker B, Hecker A, Brenck F, Preuß M, Schmelzer T, Padberg W, Weigand MA, Klasen J (2012). Early tracheostomy decreases ventilation time but has no impact on mortality of intensive care patients: a randomized study. Langenbeck's Arch Surg.

[6] Rumbak MJ, Newton M, Truncale T, Schwartz SW, Adams JW, Hazard PB (2004). A prospective, randomized, study comparing early percutaneous dilational tracheotomy to prolonged translaryngeal intubation (delayed tracheotomy) in critically ill medical patients. Crit Care Med.

[7] Terragni PP, Antonelli M, Fumagalli R, Faggiano C, Berardino M, Pallavicini FB, Miletto A, Mangione S, Sinardi AU, Pastorelli M (2010). Early vs late tracheotomy for prevention of pneumonia in mechanically ventilated adult ICU patients: a randomized controlled trial. JAMA.

[8] Trouillet JL, Luyt CE, Guiguet M, Ouattara A, Vaissier E, Makri R, Nieszkowska A, Leprince P, Pavie A, Chastre J (2011). Early percutaneous tracheotomy versus prolonged intubation of mechanically ventilated patients after cardiac surgery: a randomized trial. Ann Intern Med.

[9] Young D, Harrison DA, Cuthbertson BH, Rowan K (2013). Effect of early vs late tracheostomy placement on survival in patients receiving mechanical ventilation: the TracMan randomized trial. JAMA.

[10] Zheng Y, Sui F, Chen XK, Zhang GC, Wang XW, Zhao S, Song Y, Liu W, Xin X, Li WX (2012). Early versus late percutaneous dilational tracheostomy in critically ill patients anticipated requiring prolonged mechanical ventilation. Chin Med J.

[11] Hosokawa K, Nishimura M, Egi M, Vincent JL (2015). Timing of tracheotomy in ICU patients: a systematic review of randomized controlled trials. Crit Care (Lond, Engl).

[12] Deng H, Fang Q, Chen K, Zhang X (2021). Early versus late tracheotomy in ICU patients: a meta-analysis of randomized controlled trials. Medicine.

[13] Wang R, Pan C, Wang X, Xu F, Jiang S, Li M (2019). The impact of tracheotomy timing in critically ill patients undergoing mechanical ventilation: a meta-analysis of randomized controlled clinical trials with trial sequential analysis. Heart Lung J Crit Care.

[14] Adly A, Youssef TA, El-Begermy MM, Younis HM (2018). Timing of tracheostomy in patients with prolonged endotracheal intubation: a systematic review. Eur Arch Otorhinolaryngol.

[15] Siempos II, Ntaidou TK, Filippidis FT, Choi AMK (2015). Effect of early versus late or no tracheostomy on mortality and pneumonia of critically ill patients receiving mechanical ventilation: a systematic review and meta-analysis. Lancet Respir Med.

[16] Chorath K, Hoang A, Rajasekaran K, Moreira A (2021). Association of early vs late tracheostomy placement with pneumonia and ventilator days in critically ill patients: a meta-analysis. JAMA Otolaryngol Head Neck Surg.

[17] Andriolo BN, Andriolo RB, Saconato H, Atallah ÁN, Valente O (2015). Early versus late tracheostomy for critically ill patients. Cochrane Database Syst Rev.

[18] Bier-Laning C, Cramer JD, Roy S, Palmieri PA, Amin A, Añon JM, Bonilla-Asalde CA, Bradley PJ, Chaturvedi P, Cognetti DM (2020). Tracheostomy during the COVID-19 pandemic: comparison of international perioperative care protocols and practices in 26 countries. Otolaryngol Head Neck Surg.

[19] McGrath BA, Brenner MJ, Warrillow SJ, Pandian V, Arora A, Cameron TS, Añon JM, Hernández Martínez G, Truog RD, Block SD (2020). Tracheostomy in the COVID-19 era: global and multidisciplinary guidance. Lancet Respir Med.

[20] Ji Y, Fang Y, Cheng B, Li L, Fang X (2022). Tracheostomy timing and clinical outcomes in ventilated COVID-19 patients: a systematic review and meta-analysis. Crit Care (Lond, Engl).

[21] Tsonas AM, Botta M, Horn J, Brenner MJ, Teng MS, McGrath BA, Schultz MJ, Paulus F, Serpa Neto A (2022). Practice of tracheostomy in patients with acute respiratory failure related to COVID-19-insights from the PRoVENT-COVID study. Pulmonology.

[22] Navaratnam AV, Gray WK, Wall J, Takhar A, Day J, Tatla T, Batchelor A, Swart M, Snowden C, Marshall A (2022). Utilisation of tracheostomy in patients with COVID-19 in England: patient characteristics, timing and outcomes. Clin Otolaryngol.

[23] Pauli N, Eeg-Olofsson M, Bergquist H (2021). Tracheotomy in COVID-19 patients: a retrospective study on complications and timing. Laryngoscope Investig Otolaryngol.

[24] Ranieri VM, Rubenfeld GD, Thompson BT, Ferguson ND, Caldwell E, Fan E, Camporota L, Slutsky AS (2012). Acute respiratory distress syndrome: the Berlin Definition. JAMA.

[25] Moreno RP, Metnitz PG, Almeida E, Jordan B, Bauer P, Campos RA, Iapichino G, Edbrooke D, Capuzzo M, Le Gall JR (2005). SAPS 3–From evaluation of the patient to evaluation of the intensive care unit. Part 2: Development of a prognostic model for hospital mortality at ICU admission. Intensive Care Med.

[26] Vagionas D, Vasileiadis I, Rovina N, Alevrakis E, Koutsoukou A, Koulouris N (2019). Daily sedation interruption and mechanical ventilation weaning: a literature review. Anaesthesiol Intensive Ther.

[27] Nieszkowska A, Combes A, Luyt CE, Ksibi H, Trouillet JL, Gibert C, Chastre J (2005). Impact of tracheotomy on sedative administration, sedation level, and comfort of mechanically ventilated intensive care unit patients. Crit Care Med.

[28] Frithiof R, Rostami E, Kumlien E, Virhammar J, Fällmar D, Hultström M, Lipcsey M, Ashton N, Blennow K, Zetterberg H (2021). Critical illness polyneuropathy, myopathy and neuronal biomarkers in COVID-19 patients: a prospective study. Clin Neurophysiol Off J Int Fed Clin Neurophysiol.

[29] Huang C, Huang L, Wang Y, Li X, Ren L, Gu X, Kang L, Guo L, Liu M, Zhou X (2021). 6-month consequences of COVID-19 in patients discharged from hospital: a cohort study. Lancet.

[30] Fan E, Beitler JR, Brochard L, Calfee CS, Ferguson ND, Slutsky AS, Brodie D (2020). COVID-19-associated acute respiratory distress syndrome: is a different approach to management warranted?. Lancet Respir Med.

[31] Grieco DL, Bongiovanni F, Chen L, Menga LS, Cutuli SL, Pintaudi G, Carelli S, Michi T, Torrini F, Lombardi G (2020). Respiratory physiology of COVID-19-induced respiratory failure compared to ARDS of other etiologies. Crit Care (Lond, Engl).

[32] Ziehr DR, Alladina J, Petri CR, Maley JH, Moskowitz A, Medoff BD, Hibbert KA, Thompson BT, Hardin CC (2020). Reply to Yaroshetskiy et al.: Acute respiratory distress syndrome in COVID-19: do all these patients definitely require intubation and mechanical ventilation?. Am J Respir Criti Care Med.

[33] Chiumello D, Busana M, Coppola S, Romitti F, Formenti P, Bonifazi M, Pozzi T, Palumbo MM, Cressoni M, Herrmann P (2020). Physiological and quantitative CT-scan characterization of COVID-19 and typical ARDS: a matched cohort study. Intensive Care Med.

