# Supplementary material for: TTCOV19: timing of tracheotomy in SARS-CoV-2-infected patients: a multicentre, single-blinded, randomized, controlled trial
Source: Crit Care. 2022 May 18;26:142. doi: 10.1186/s13054-022-04005-0 (PMC9115544; doi:10.1186/s13054-022-04005-0)
Supplement: Supplementary file 1 — Additional file 1. TTCOV19 Research Protocol. [file 13054_2022_4005_MOESM1_ESM.docx]

**Research protocol**

Title: **Timing of Tracheotomy in Covid-19-positive patients (TTCOV19) – a randomized controlled trial.**

Date: 18^th^ of May 2020

**Researchers involved**: Måns Eeg-Olofsson1, Nina Pauli1, Louise Hafsten1, Christopher Lundborg2, Karin Löwhagen2, Katarina Hallén2, Magnus Brink3, Magnus Gisslén3 and Henrik Bergquist1.

ENT clinic1, Anesthesia and intensive care clinic2 and Clinic of infectious diseases3, Sahlgrenska University Hospital, Gothenburg

**Background:** The SARS-Cov-2 virus and its associated disease Covid-19 are spreading worldwide, and with it an increasing need for intubation and ventilator care of the most severely affected patients (1). In particular, the development of the inflammatory lung disease Acute Respiratory Distress Syndrome (ARDS) is causing the latter. It has been known for some time that prolonged intubation can cause damage to the trachea, which is why a tracheotomy is performed after a period of time, i.e. an opening is surgically created in the trachea to allow continued ventilator care via a tracheal cannula. A tracheotomy may also reduce the need for sedation, facilitate withdrawal from the ventilator, reduce the need for ICU care and possibly reduce the risk of bedsores, thromboembolism and death (2). However, when in the time course it is optimal to perform a tracheotomy in covid 19 patients is not known but can hopefully be clarified with the current study (3,4).

**Aim/Purpose:** The aim of the study is to find out if the time aspect from intubation to tracheotomy affects the outcome of the patient's health during the continued ICU care. Further aims are to find out how underlying risk factors including cardiovascular and pulmonary disease affect outcome and to identify all cases intubated due to covid-19 infection. The latter will be done both retrospectively and prospectively.

**Main hypothesis:** Early tracheotomy in patients intubated due to covid-19 infection results in fewer days requiring mechanical ventilation (primary endpoint) compared to later tracheotomy.

**Materials and methods**: All patients in the Västra Götaland region who are intubated due to covid-19-induced respiratory insufficiency will be included in the study. Patient data according to CRF 1 (Appendix 1a) will be collected and completed by the appropriate ICU physician and if no exclusion criteria are present (age <18 years, expected need for ventilator therapy <14d, tracheotomy not possible within 7 days due to patient's general condition, tracheotomy not possible due to abnormal anatomy, no consent from patient or family/next of kin), the patient will be included in the study and proceed to randomization either with the intention to undergo early tracheotomy (within 1 week after intubation) or late tracheotomy (after at least 10 days of intubation), see flowchart (Figure 1). Data from a retrospective analysis of the patients who have been tracheotomized so far due to Covid-19 disease will be used to obtain information on specific risk factors so that this can be taken into account at randomization.

Likely risk factors are cardiovascular and pulmonary disease, old age, gender, BMI, smoking and diabetes. Randomization will be done via a data algorithm with blocking to ensure equal distribution between treatment arms in terms of risk factors. A research nurse oversees the randomization process, assigns patients a study number and obtains the CRF 1.

Deviation from the treatment strategy provided by the randomization may occur if the clinical course suggests that this would be most beneficial for the individual patient. At the time of discharge from the ICU, the CRF 2 (Appendix 1b) is completed by the ICU physician in question, who sends it to the research nurse. The information from CRF 1 and 2 is entered into a database and random checks of the data are performed to ensure validity. In addition to the information from CRF 1 and 2, any mortality within 90 days of intubation will be recorded and included as part of the analysis. The analysis will be both intention-to-treat and per-protocol, with the primary outcome measure being the number of days on ventilator and the other parameters included in CRF 1 and 2, including underlying risk factors, being secondary. The study will be single-blinded, i.e. the patient will not know the randomization result until at least after the end of the study, but the treating physician will be aware of it.

TTCOV19

Follow-up

Follow-up

Death

Surg≥10 d PP

Surg≤7d d

PP

ITT

Death

Death

ITT

ITT Trach≤7d dd

Randomization

Exclusion

Death

Inclusion

Follow-up

Surg 8-9 d

Trach≥10 d

Fig 1. Flowchart of the TTCOV19 studies. All patients intubated due to Covid-19 infection are included. Exclusion is according to CRF 1. Randomization to tracheotomy within 7 days or at least 10 days after intubation. Follow-up according to CRF 2. Trak = intention to perform tracheotomy. Surg = surgery performed. ITT = Intention-to-treat analysis. PP = Per-protocol analysis

**Statistics:** Based on previous studies with the same primary outcome measure (number of days requiring ventilator care), approximately 70 evaluable patients (per-protocol analysis) in each treatment arm are required to achieve 80% power with a significance level of p<0.05 (4-6). We anticipate that approximately 20% of patients will not receive tracheotomy according to randomization results for various reasons and will instead be analyzed according to intention-to-treat. This implies that we expect to need to include about 90 patients in each treatment arm. An interim analysis will be performed when half the number of patients in each arm are included and evaluable. Mann Whitney U-test (non-parametric) will be used to compare both the primary and secondary outcome measures (categorical data) between treatment arms.

**Time aspect of the study implementation:** We expect to be able to start inclusion during May/June 2020. Based on the number of patients who have been tracheotomized in the Gothenburg region so far, inclusion in the study is expected to last until November 2020, but this time period may be significantly shortened depending on how many other hospitals in the VG region choose to participate. Interim analysis is expected to be performed in August/September 2020 at the earliest. Analysis of final data is expected in December/January 2020/21 with presentation and submission to a scientific journal in early 2021. The study will be registered on ClinicalTrials.gov.

**References:**

- 1. Phua, J et al. Intensive care management of coronavirus disease 2019 (COVID-19): challenges and recommendations. Lancet Respir Med 2020 Apr 6;S2213- 2600(20)30161-2
  2. Marini, J and Gattinoni, L. Management of COVID-19 Respiratory Distress. JAMA 24 April 2020.
  3. Cochrane Evidence. 12 Jan 2015. Timing of tracheostomy for critically ill patients who are predicted to be on long-term artificial respiration.
  4. Diaz-Prieto, et al. A randomized clinical trial for the timing of tracheotomy in critically ill patients: factors precluding inclusion in a single center study. Crit Care. 2014 Oct 29;18(5):585.
  5. Dochi et al., Effect of Early Tracheostomy in Mechanically Ventilated Patients. Laryngoscope Investig Otolaryngol. 2019 Apr 22;4(3):292-299.
  6. Zheng et al., Early Versus Late Percutaneous Dilational Tracheostomy in Critically Ill Patients Anticipated Requiring Prolonged Mechanical Ventilation. Chin Med J (Engl). 2012 Jun;125(11):1925-30.

Appendix 1a: CRF 1

Appendix 1b: CRF 2

**Case Report Form 1 TTCOV19 study**

(to be completed on all patients intubated for Covid-19 infection)

◻ Name and social security number: ____________________________

◻ Date of confirmed Covid-19 infection: ___________________________

◻ Date of symptom onset:________ Date of intubation:_____________

◻ Reason for intubation: _______________________________

______________________________________________________

◻ Death during/after intubation, date:

◻ BMI:

◻ Heart disease: ◻Hypertension ◻Smoking

◻ COPD ◻Asthma ◻Diabetes

◻OSAS

◻ Other significant comorbidity^2^:

___________________________________________________________

◻ Current medications:

_____________________________________________________________

◻ Any reason for exclusion^3^:

◻ Consultation with patient or family/good man regarding study participation

◻ Research nurse Louise Hafsten (tel 070-4885456) contacted for study number and randomization (kept on file)

◻ Study number: randomization (7d/10d):

◻ Referral written to ENT regarding intention of early surgical tracheotomy

**Case Report Form 2 TTCOV19 study**

(to be filled in at discharge from IVA, sent to research nurse Louise Hafsten)

◻ Study number: ◻randomization (7d/10d):___________________

◻ Time intubation - tracheotomy: days_________________________

◻ Reason for not performing tracheotomy according to randomization:

_______________________________________________________________________

◻ Surgical alt percutaneous tracheotomy: ____________________________________

◻ Type of tracheal cannula initially: _________________________________________

◻ Total number of days on ventilator (primary endpoint): ______________________

◻ Total number of days with sedation: _____________________________________

◻ Total number of days with supplemental oxygen requirement: _________________

◻ Total number of days with ventilation in prone position: ___________________________

◻ Total number of days in ICU: ______________________________________________

◻ Medication (covid-specific): ________________________________________

◻ Complications during ICU care^1^: _________________________________________

◻ Need for reintubation, reason:___________________________________________

◻ Confirmed infection by staff who cared for the patient, how: __________________

◻ Death in IVA, cause: ________________________________________________________
